# Posterior circulation stroke: machine learning-based detection of early ischemic changes in acute non-contrast CT scans

**DOI:** 10.1007/s00415-020-09859-4

**Published:** 2020-05-11

**Authors:** Helge C. Kniep, Peter B. Sporns, Gabriel Broocks, André Kemmling, Jawed Nawabi, Thilo Rusche, Jens Fiehler, Uta Hanning

**Affiliations:** 1 Department of Diagnostic and Interventional Neuroradiology, University Medical Center Hamburg-Eppendorf, Martinistr. 52, 20246 Hamburg, Germany; 2 Institute of Clinical Radiology, University Hospital of Muenster, Albert-Schweitzer-Campus 1, 48149 Muenster, Germany; 3 Department of Neuroradiology, University Medical Center Schleswig-Holstein, Ratzeburger Allee 160, 23562 Lübeck, Germany; 4 Department of Radiology, Charité University Medical Center Berlin, Charitéplatz 1, 10117 Berlin, Germany

**Keywords:** Stroke, Basilar artery occlusion, CT imaging, Early infarct signs, Machine learning, Computer-assisted radiographic image interpretation

## Abstract

**Objectives:**

Triage of patients with basilar artery occlusion for additional imaging diagnostics, therapy planning, and initial outcome prediction requires assessment of early ischemic changes in early hyperacute non-contrast computed tomography (NCCT) scans. However, accuracy of visual evaluation is impaired by inter- and intra-reader variability, artifacts in the posterior fossa and limited sensitivity for subtle density shifts. We propose a machine learning approach for detecting early ischemic changes in pc-ASPECTS regions (Posterior circulation Alberta Stroke Program Early CT Score) based on admission NCCTs.

**Methods:**

The retrospective study includes 552 pc-ASPECTS regions (144 with infarctions in follow-up NCCTs) extracted from pre-therapeutic early hyperacute scans of 69 patients with basilar artery occlusion that later underwent successful recanalization. We evaluated 1218 quantitative image features utilizing random forest algorithms with fivefold cross-validation for the ability to detect early ischemic changes in hyperacute images that lead to definitive infarctions in follow-up imaging. Classifier performance was compared to conventional readings of two neuroradiologists.

**Results:**

Receiver operating characteristic area under the curves for detection of early ischemic changes were 0.70 (95% CI [0.64; 0.75]) for cerebellum to 0.82 (95% CI [0.77; 0.86]) for thalamus. Predictive performance of the classifier was significantly higher compared to visual reading for thalamus, midbrain, and pons (*P* value < 0.05).

**Conclusions:**

Quantitative features of early hyperacute NCCTs can be used to detect early ischemic changes in pc-ASPECTS regions. The classifier performance was higher or equal to results of human raters. The proposed approach could facilitate reproducible analysis in research and may allow standardized assessments for outcome prediction and therapy planning in clinical routine.

**Electronic supplementary material:**

The online version of this article (10.1007/s00415-020-09859-4) contains supplementary material, which is available to authorized users.

## Introduction

Ischemic stroke in the posterior circulation (pc) due to basilar artery occlusion is frequently associated with poor outcome. Efficient triage of patients for additional imaging diagnostics, adequate therapy regimes, and initial outcome prediction requires detection of early ischemic changes in early hyperacute non-contrast computed tomography (NCCT) scans [24]. However, accuracy of visual assessments of admission NCCT images is impaired by inter- and intra-reader variability, artifacts, and reduced imaging quality in the posterior fossa. Furthermore, subtle early ischemic changes might not be detectable by human eyes.

A promising approach to overcome these limitations is an automated machine learning-based interpretation of quantitative radiomic image features extracted from pre-therapeutic NCCT scans at admission. NCCT scans at admission are fast and the required equipment is broadly available. Furthermore, NCCT imaging is a fundamental part of most standard-of-care stroke protocols. Such diagnostic tool could (a) facilitate standardized evaluation of early ischemic changes at improved sensitivity in clinical routine and large-scale clinical studies, (b) increase precision of triage for additional MR imaging diagnostics, therapy planning and outcome prediction, and hence (c) improve patient care at low risk and cost using readily available admission NCCT scans.

We hypothesized that quantitative radiomic image features extracted from early hyperacute NCCT brain scans can be used to detect early ischemic changes in posterior circulation Alberta Stroke Program Early CT Score (pc-ASPECTS) regions. pc-ASPECTS encompasses clinically relevant areas of the posterior circulation and has been demonstrated to allow for an objective prediction of the patient’s prognosis [13, 21, 24, 28].

We employed a previously published and established radiomics machine learning pipeline on NCCT brain scans of patients with basilar artery occlusion [6, 26] undergoing successful recanalization. Furthermore, we evaluated performance of the proposed algorithm in comparison to conventional visual assessments of two neuroradiologist readers.

## Materials and methods

This single center retrospective study was approved by the Ethics Committee of the University of Muenster, and the requirement for informed consent was waived (2017-233-f-S). All study protocols and procedures were conducted in accordance with the Declaration of Helsinki. The data used for training and validation of algorithms in this study are available from the corresponding author upon reasonable request.

**Fig. 1:**
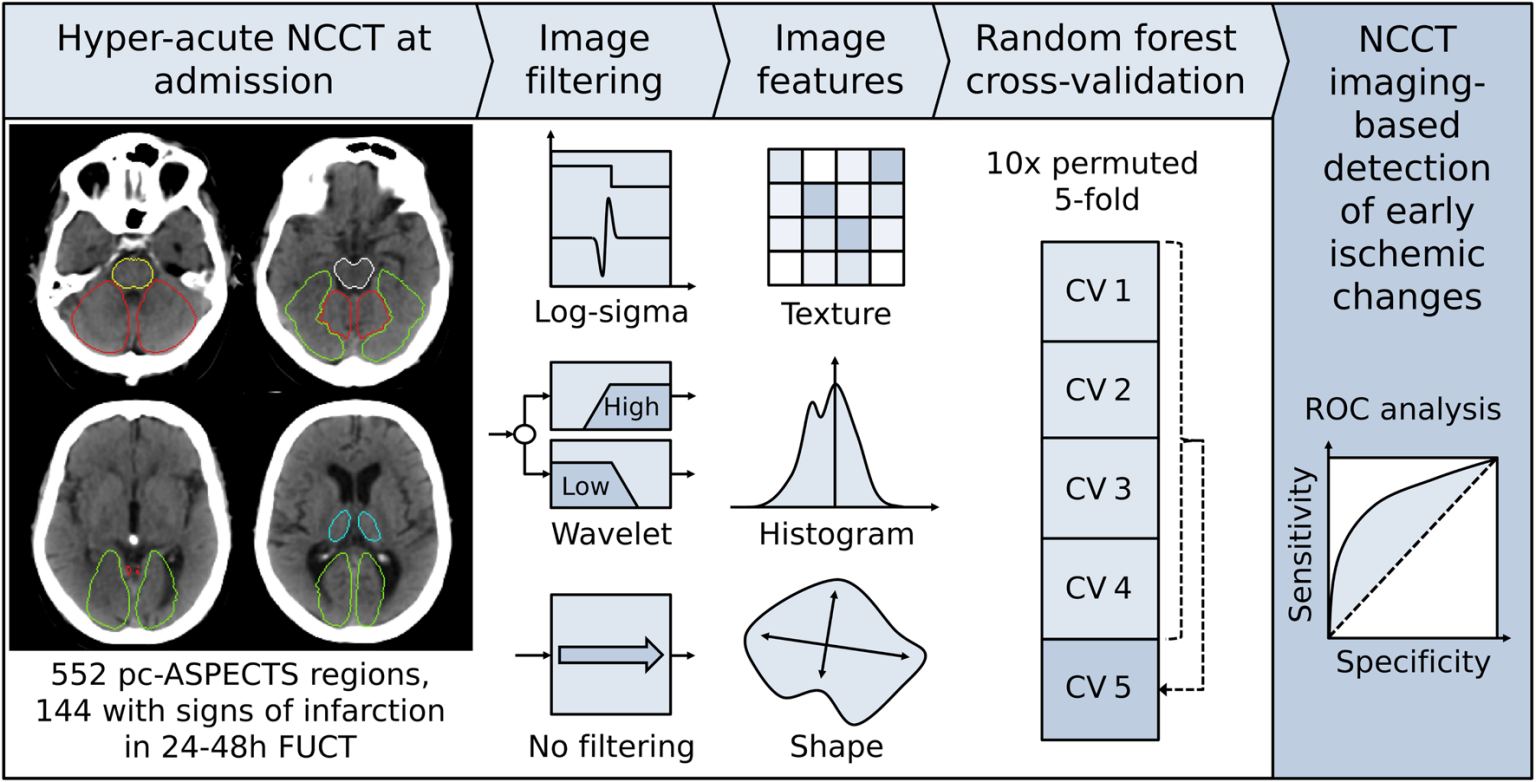
Conceptual overview of the proposed machine learning approach showing the major processing steps: CT-based image acquisition and segmentation, feature extraction (*n* = 1218), and statistical learning (random forest algorithm). *NCCT* non-contrast computed tomography, *pc-ASPECTS* Posterior circulation Acute Stroke Prognosis Early CT Score, *FUCT* follow-up computed tomography, *PCA* posterior cerebral artery

An overview of the proposed approach is given in Fig. 1; its modules are detailed below.

### Patients

The study cohort includes consecutive patients with basilar artery occlusion admitted between January 1, 2011, and March 31, 2017 at a tertiary care stroke center. The inclusion criteria for this study were (a) acute basilar artery occlusion; (b) successful recanalization (mTICI ≥ 2b); (c) NCCT performed on admission; and (d) NCCT performed < 6 h after symptom onset (early hyperacute). Exclusion criteria were (a) poor imaging quality; (b) not all regions of the posterior circulation included in the NCCT images and (c) missing follow-up imaging.

### Image acquisition

NCCT imaging at admission was performed within a time window of up to 6 h after symptom onset (hyperacute). Follow-up imaging consisted of NCCT 24–48 h after admission or earlier if clinically relevant. NCCT scans were acquired on a 128-slice dual-source CT scanner (Somatom Definition Flash; Siemens Healthcare GmbH). NCCT head images were obtained from the vertex to the skull base (120 kV, 340 mAs, 5.0 mm slice reconstruction, < 0.5 mm in-plane resolution, 1.0 mm increment, 0.6 mm collimation, 0.8 pitch and H30s soft kernel).

### Registration to standard space

To extract information from standardized pc-ASPECTS maps and to reduce potential bias in quantitative texture analysis, all NCCT images were registered to a custom MNI-152 CT reference image [5] using two-step affine algorithms [14]. Registration success was visually verified by two MDs (UH and PS: 8 years of clinical experience in diagnostic neuroradiology in acute care full-service hospitals).

### pc-ASPECTS maps

Standardized pc-ASPECTS area maps [thalamus left/right (l/r), pons, midbrain, territory of the posterior cerebral artery (PCA) l/r, cerebellum l/r] were derived as follows: First, an experienced neuroradiologist (UH) performed manual segmentations of the respective regions on the original NCCT images of the 63 healthy subjects using Analyze 11.0 Software (Biomedical Imaging Resource, Mayo Clinic, Rochester, MN) [2]. Second, manual segmentations were transformed into standard space by employing transformation matrices and control point grids obtained from image registration to the custom MNI-152 CT reference image [5]. Third, all segmentations were added and final standard maps were defined using median cut-off points.

### Ground truth

Infarct lesion assessment in follow-up NCCT images is used as widely accepted imaging endpoint in major clinical trials. Accordingly, for all pc-ASPECTS regions that showed clear signs of final infarction in follow-up NCCT imaging, the occurrence of early ischemic changes on admission NCCT images was assumed. Two senior MDs (UH, PS) rated infarction (early ischemic changes expected in early hyperacute NCCT images) or no infarction (no early ischemic changes expected in early hyperacute NCCT images) for each pc-ASPECTS region through conventional reading of the follow-up NCCT images. Both readers were blinded to any further clinical information or imaging data. Conflicts in classification were solved in a consensus reading process.

### Feature extraction

Quantitative image features were extracted using the PyRadiomics Python package v2.1.0 [26], proposed default settings were used for the analysis. Extracted features comprised 252 first-order features (18 based on unfiltered images, 144 wavelet decompositions, 90 log-sigma Laplacian of Gaussian filtered images) and 966 texture features (82 based on unfiltered images, 544 wavelet decompositions, 340 log-sigma Laplacian of gaussian filtered images). In total, 1218 quantitative image features were extracted from each of the 552 included pc-ACPECTS areas.

### Machine learning

Machine learning-based detection of early ischemic changes was performed using random forest algorithms (Python scikit-learn environment v0.20.3 [17]). Random forest classifiers have a comparably low tendency to overfit [3] and support classification tasks also for data sets comprising numerous and heterogeneous predictors. It was demonstrated that random forest algorithms can achieve stable results based on standard hyperparameter settings and are comparably insensitive to further parameter tuning [19]. Recent analyses report a maximum gain in areas under the curve (AUCs) of 0.007 after hyperparameter tuning vs. common standard settings for 39 different test data sets [20]. To ensure maximum generalizability of the proposed algorithm and to avoid overfitting with respect to parameter tuning, random forest classifier settings were defined a priori according to established standard parameter values for classification tasks [3, 19]. The number of trees was set to a comparably high value of 1000 to allow for sufficient precision in variable importance estimation. Following established recommendations for classification tasks, the maximum number of split variables per node (*m*_try_) was limited to the square root of the total number of features. Tuning of tree complexity, sampling scheme and splitting rules were shown to have only marginal impact on classification performance [20] and were kept at the scikit-learn default settings. To account for differences of quantitative image features caused by the different anatomical locations, separate classifiers were trained for each considered region. However, bilaterally equal regions (e.g., thalamus l/r) were analyzed with the same classifiers. To prevent overestimation of classification performance due to effects from cluster correlation, bilateral regions of the same patient were either assigned to the training or the validation set. Due to the relatively balanced dataset with c. 30% event rate (144/512), no additional data augmentation for reducing bias from class imbalance was performed.

### Model performance assessment

Conformable to established evaluation procedures, model performance was tested using fivefold cross-validation with independent training and validation sets in a model-external approach [8]. Stability of predictive performance was assessed through comparative analysis of ten randomly permuted fivefold cross-validation sets.

### Feature selection

Image features were sorted according to predictive value based on Gini impurity measures [9] for each training data set. To improve generalizability and to reduce bias from potentially unimportant features in final model training and performance testing, the number of employed predictors was limited to the 20 most important features of each respective training data set.

### Neuroradiologist reading

Two experienced neuroradiologists visually detected early ischemic changes on the hyperacute NCCT images. For each pc-ASCPECTS region, the readers rated “early ischemic changes” or “no early ischemic changes”. Both readers were blinded to the ground truth, the classifier prediction and the other reader’s prediction.

### Statistical analysis

Receiver operating characteristic (ROC) curves for per-region detection of early ischemic changes were derived based on predictions performed on the validation sets. Statistical significance of AUCs was assumed if *P* value < 0.05 with H0: AUC = 0.50 (random guess) for all validation sets. Model prediction instability (i.e. standard deviation of AUCs) was evaluated using ten randomly drawn fivefold cross-validation sets. *P* values were calculated according to Mann–Whitney/Wilcoxon *U* statistics using the verification v1.42 R-package [11]. Confidence intervals for sensitivities and specificities were derived using pROC v1.10 [22] and DTComPair v1.0.3 R-packages. Generalized classification performance for per-region detection of early ischemic changes of neuroradiologist readers and the machine learning classifier was compared using Matthews correlation coefficient (MCC) [12]. MCC evaluates all fields of the confusion matrix and is considered as a favorable metric for unbiased comparisons of binary classifiers [18]. With *TP*: true positives, *TN*: true negatives, *FP*: false positives, and *FN*: false negatives MCC is defined as:

$${\text{MCC}} = \frac{{{\text{TP}} \times {\text{TN}} - {\text{FP}} \times {\text{FN}}~}}{{\sqrt {\left( {{\text{TP}} + {\text{FP}}} \right)\left( {{\text{TP}} + {\text{FN}}} \right)\left( {{\text{TN}} + {\text{FP}}} \right)\left( {{\text{TN}} + {\text{FN}}} \right)} }}.$$

MCC confidence intervals were computed with the psychometric v2.2 R-package. Statistical significances of differences in MCC were calculated using the psych v1.8.12 R-package.

## Results

The analysis is based on NCCT images of 552 pc-ASPECTS regions extracted from 69 patients (37 females, median age 74 year, IQR 60–80 years) with acute basilar artery occlusion, thereof 144 regions with definite infarction in the follow-up imaging and 408 regions without infarction (Table 1). Median NIHSS score was 11 (interquartile range 4–17), all patients underwent successful recanalization (mTICI ≥ 2b), 42 patients (60.9%) were treated with IV thrombolysis (Table 1).

**Table 1:** Baseline characteristics of the study patients

Patient characteristics	*n* = 69
Age at admission (year) [median (IQR)]	74 (60; 80)
Female *n* (%)	37 (52.1)
Baseline NIHSS [median (IQR)]	11 (4; 17)
Intravenous thrombolysis [*n* (%)]	42 (60.9)
Diabetes mellitus [*n* (%)]	22 (31.9)
Hypercholesterolemia [*n* (%)]	18 (26.1)
Arterial hypertension [*n* (%)]	50 (72.5)
Arterial fibrillation [*n* (%)]	30 (43.5)
Smoking [*n* (%)]	8 (11.6)
Etiology atherosclerosis [*n* (%)]	32 (46.6)
Etiology cardioembolism [*n* (%)]	7 (10.1)
Etiology other [*n* (%)]	1 (1.4)
Etiology unknown [*n* (%)]	29 (42)

pc-ASPECTS regions	*n* = 552
Cerebellum [*n* (*n* with infarction in FUCT)]	138 (41)
Midbrain [*n* (*n* with infarction in FUCT)]	69 (21)
PCA [*n* (*n* with infarction in FUCT)]	138 (33)
Pons [*n* (*n* with infarction in FUCT)]	69 (24)
Thalamus [*n* (*n* with infarction in FUCT)]	138 (25)
Total [*n* (*n* with infarction in FUCT)]	552 (144)
Number of regions with infarction in FUCT per patient [median (IQR)]	1 (0; 3)

*NIHSS* National Institutes of Health Stroke Scale, *FUCT* follow-up non-contrast computed tomography, *pc-ASPECTS* Posterior circulation Alberta Stroke Program Early CT Score, *FU* follow-up, *PCA* posterior cerebral artery

### Classifier performance

**Fig. 2:**
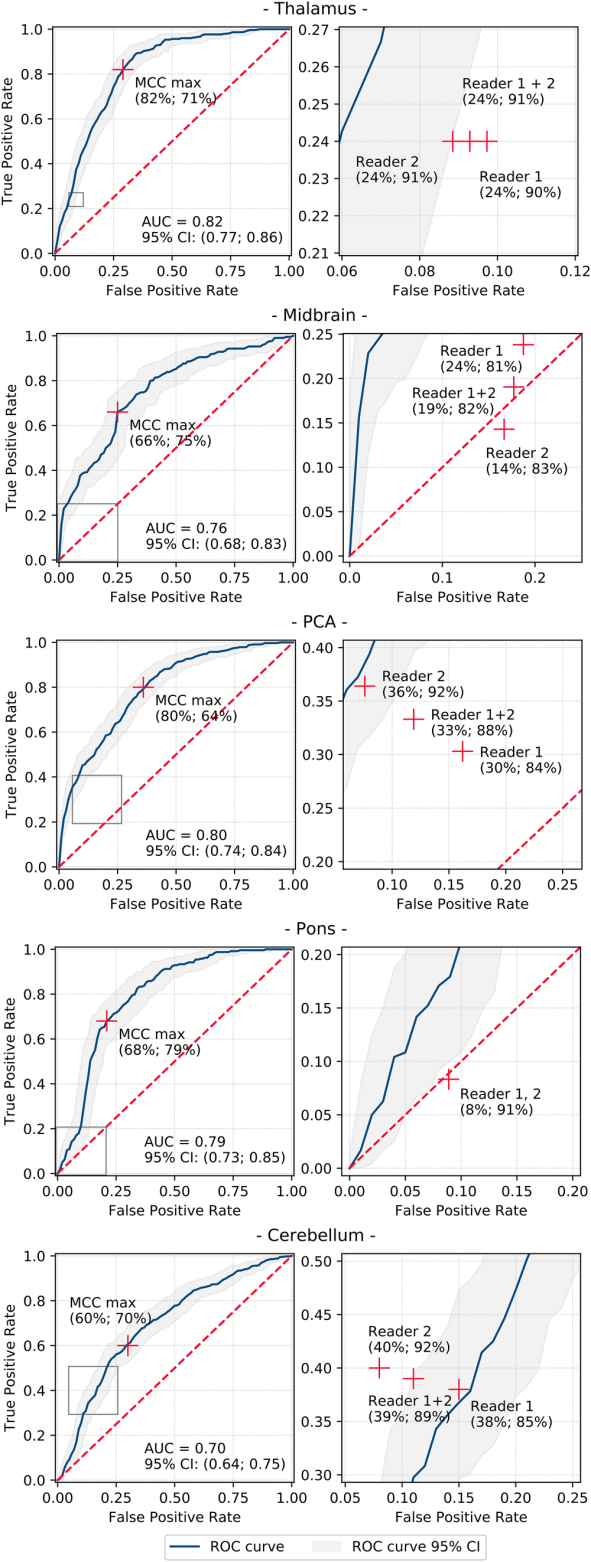
Receiver operating characteristic curves and neuroradiologist readings operating points for detecting early ischemic changes in pc-ASPECTS regions based on acute NCCT scans. Left graphs: machine learning classifier receiver operating characteristic (ROC) curves with optimal operating point at maximum MCC (sensitivity; specificity), grey rectangles define cut-out areas shown in graphs on the right; right graphs: cut-outs of left figures showing neuroradiologist reader rating results (sensitivity; specificity). Blue lines depict ROC curves, grey areas shows 95% confidence intervals (CI). Red crosses show cut-off points/prediction performance. *AUC* area under the curve, *CI* confidence interval, *ROC* receiver operating characteristics, *NCCT* non-contrast computed tomography, *pc-ASPECTS* Posterior circulation Acute Stroke Prognosis Early CT Score, *MCC* Matthews correlation coefficient, *PCA* posterior cerebral artery

ROC AUCs in the validation sets for per-region detection of early ischemic changes were 0.70 for cerebellum to 0.82 for thalamus (Fig. 2). At maximum MCC cut-off values, the classifiers yielded specificities of 64–79% at sensitivities of 60–82% (Fig. 2, Table 2).

**Table 2:** Per-region classification performance for detection of early ischemic changes

Region	Prediction	Cut-off point	Sensitivity (95% CI)	Specificity (95% CI)	Accuracy (%)	Youden index	MCC (95% CI)
Thalamus	Reader 1 + 2	–	24% (12%; 36%)	91% (87%; 94%)	79	0.15	0.17 (0.00; 0.32)
	Classifier	Specificity R1 + R2	39% (33%; 45%)	91% (89%; 92%)	80	0.30	0.33* (0.17; 0.47)
	Classifier	Maximum MCC	82% (78%; 87%)	71% (68%; 73%)	73	0.53	0.44** (0.29; 0.57)
Midbrain	Reader 1 + 2	–	19% (7%; 31%)	82% (75%; 90%)	63	0.01	0.02 (− 0.22; 0.25)
	Classifier	Specificity R1 + R2	48% (41%; 54%)	82% (78%; 85%)	71	0.29	0.30* (0.07; 0.50)
	Classifier	Maximum MCC	66% (60%; 73%)	75% (71%; 78%)	72	0.41	0.39** (0.17; 0.57)
PCA	Reader 1 + 2	–	33% (22%; 45%)	88% (84%; 92%)	75	0.21	0.24 (0.08; 0.39)
	Classifier	Specificity R1 + R2	47% (41%; 52%)	88% (87%; 91%)	79	0.35	0.38 (0.23; 0.51)
	Classifier	Maximum MCC	80% (76%; 85%)	64% (61%; 67%)	68	0.44	0.38* (0.23; 0.51)
Pons	Reader 1 + 2	–	8% (1%; 16%)	91% (85%; 97%)	62	− 0.01	− 0.01 (− 0.23; 0.25)
	Classifier	Specificity R1 + R2	26% (21%; 32%)	91% (87%; 92%)	68	0.16	0.21* (0.00; 0.43)
	Classifier	Maximum MCC	68% (62%; 74%)	79% (75%; 83%)	75	0.47	0.46** (0.25; 0.63)
Cerebellum	Reader 1 + 2	–	39% (30%; 51%)	89% (84%; 93%)	74	0.28	0.32 (0.16; 0.46)
	Classifier	Specificity R1 + R2	30% (26%; 35%)	89% (87%; 91%)	71	0.19	0.23 (0.07; 0.38)
	Classifier	Maximum MCC	60% (56%; 65%)	70% (67%; 73%)	67	0.31	0.29 (0.12; 0.44)

Classifier metrics are shown at cut-off points according to neuroradiologist readers’ specificities and at the classifiers optimal operating point

*MCC* Matthews correlation coefficient, *CI* confidence interval, *PCA* posterior cerebral artery

*P value < 0.05; **P value < 0.01. *P* values refer to significance of difference between classifier and human readers MCC values

### Feature importance

**Fig. 3:**
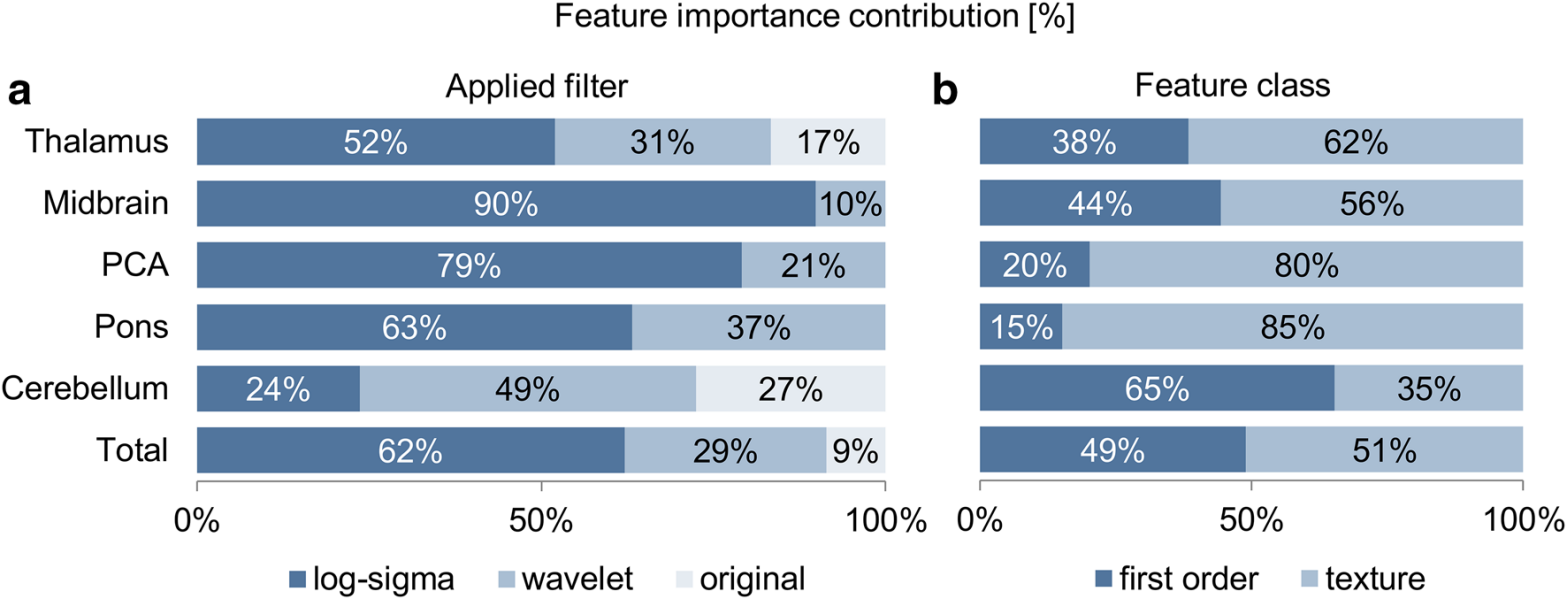
Feature importance contribution of employed 20 most important features in %. **a** By applied filter and pc-ASPECTS region; **b** by feature class and pc-ASPECTS region. Texture feature class includes gray level size zone matrix, gray level dependence matrix, gray level run length matrix and gray level co-occurrence matrix. *ROI* region of interest, *PCA* posterior cerebral artery

Feature importance analysis was conducted using mean importance values calculated across all training sets. For all pc-ASPECTS regions, top-20 features with highest importance for prediction were derived from first order and texture features at equal proportions (49% vs 51% contribution). However, per-region analysis shows significant differences with shares of texture features reaching from 35% for cerebellar infarctions to 85% for pontine infarctions (Fig. 3b). Differentiation by applied filter (Fig. 3a) indicates that unfiltered original images contribute the lowest share (9%) and log-sigma filtered images the highest share (62%) of total predictive power. Also for applied filters, results vary per region: whereas detection of ischemic changes in the cerebellar region depends on original (27%), wavelet (49%), and log-sigma (24%) filtered images, changes in the midbrain are mainly determined by log-sigma filtered images (90%). For 52 out of the 100 most important features (top-20 for 5 pc-ASPECTS regions), normalized feature values were significantly different for ischemic changes vs. no changes (*P* values < 0.5; Supplemental Table 1).

### Neuroradiologist reading

Both readers detected early ischemic changes at high specificity and low sensitivity for all analysed pc-ASPECTS regions (Fig. 2, Table 2). The combined human rating performance (reader 1 + reader 2) was highest for cerebellar infarctions (sensitivity = 39%, specificity = 89%, MCC = 0.32) and lowest for pontine (sensitivity = 8%, specificity = 91%, MCC = 0.0) and midbrain infarction (sensitivity = 19%, specificity = 82%, MCC = 0.0). MCC metrics and ROC-plot operating points indicate that human readers did not exceed the level of random prediction for pons and midbrain.

### Comparison of classifier and neuroradiologist reader detection sensitivity

Analysis at the reader’s specificity set points suggests that classification performance of the machine learning algorithms was equal or superior for all evaluated metrics in thalamic, midbrain, PCA, and pontine detection of ischemic changes (Table 2). Statistical significance of differences in MCCs was observed for thalamic (*P* value = 0.04), midbrain (*P* value = 0.01), and pontine (*P* value = 0.03) regions. For cerebellar infarctions, the classifier’s results were inferior to human readers (MCC = 0.23 vs. 0.32); however, the difference was not significant (*P* value = 0.16). At the classifier’s maximum MCC operating point, prediction performance was significantly superior (*P* value < 0.05) compared to human reader predictions in all pc-ASPECTS regions except for cerebellar infarctions (MCC of 0.29 vs. 0.32, *P* value = 0.56).

## Discussion

Machine learning-based detection of early ischemic changes was significantly superior (three out of five regions) or statistically equal (two out of five regions) compared to conventional readings of two neuroradiologists.

The proposed classifier yielded AUCs from 0.70 (cerebellum) to 0.82 (thalamus). Observed narrow confidence intervals and low standard deviations of AUCs across all validation sets indicate high stability of the predictive performance. Compared to visual ratings of the two neuroradiologist readers, the classifier’s per-region predictive performance (MCC metrics) was significantly higher (*P* value < 0.05) in thalamus, midbrain, and pons. For PCA territory, MCC metric of the classifier was higher (0.38 vs. 0.24) but did not reach statistical significance (*P* value = 0.06). Likewise, there was no significant difference between human and classifier predictions for the cerebellar region.

Although numerous interrelations between quantitative image features and clinical diagnoses have been demonstrated [29], radiomics-based machine learning approaches are still considered as black boxes with potentially irreproducible and unintelligible decision paths. The missing clear link between quantitative image features, traditional image findings, and the underlying pathology is a major point of criticism [29]. To address these concerns, we analyzed the employed image features regarding possible interpretations for visual assessment and established ties to conventional image readings: In clinical practice, ratings rely on visual detection of hypodensities as imaging marker of early ischemic changes. In thalamic infarctions, normalized feature values suggest hypodensity as predictive marker in the original as well as in the wavelet low-pass filtered images. This corresponds to typical thalamic hypodensity as early ischemic change. For other regions, classification depends more on intra-image distributions of intensities. High feature values in histogram metrics of log-sigma filtered images for pontine infarctions indicate pronounced intensity edges as predictive markers. Similar characteristics of features can be observed for PCA and cerebellar regions: measures of intensity histogram width derived from original and low-pass filtered images are important predictors of early ischemic changes. However, predictive performance of all regions also depends on texture and histogram metrics that cannot be interpreted in a visual context. This presents a clear advantage of the machine learning classifier compared to conventional visual assessments.

Performance of the classifiers was in similar ranges for all regions except for cerebellum. Also with respect to employed predictors, the cerebellar region deviates from general observations: classifiers for cerebellar ischemic changes utilize the highest share of histogram-based predictors and the lowest share of texture markers. Furthermore, the lowest importance of log-sigma-based features was observed. The findings indicate that cerebellar image textures are difficult to interpret for radiomics-based classifiers. Potential factors that might distract the algorithm are (a) texture findings in the cerebellar region that are typically dominated by folia arranged in finely spaced parallel grooves and (b) adverse imaging conditions in the posterior cranial fossa. In contrast to the proposed machine learning algorithm, human readers yielded highest prediction performance metrics for cerebellar infarctions. Hence, especially for cerebellar lesions, optimized diagnosis requires collaborative decision processes and may suggest symbiotic working models integrating both human and artificial intelligence.

Previous studies have demonstrated feasibility and reliability of automated detection of anterior circulation strokes and machine learning-based anterior circulation ASPECT scores [10, 15], leading to various commercially available tool that aid decision-making of stroke physicians. In the posterior circulation, beam-hardening artifacts significantly complicate the detection of early ischemic changes for the human visual system. However, to our knowledge, there are no other studies investigating the impact of machine learning on diagnostic performance in the posterior fossa yet.

Our study had general limitations, typically associated with quantitative radiomics-based image analysis and classification [1, 4, 7]: Differences in image acquisition settings (e.g. size of the field of view, gantry tilt), under- or overfitting of machine learning algorithms and ground truth misclassifications. These limitations could distort classification and may reduce generalizability results. Bias of these factors was minimized through (a) employment of NCCT scans acquired by the same scanner, (b) the application of random forest algorithms that are comparably stable with regards to overfitting and (c) usage of consensus ratings of two experienced neuroradiologists for ground truth definition. The risk of overfitting was further reduced by employing previously described standard hyperparameter settings [3, 19] and by evaluating multiple different models in an iterative fivefold cross-validation approach. Study-specific limitations were as follows: first, we only included a limited number of patients in a retrospective analysis; test sets did not include any independent samples from other centers. An expansion of sample size in a prospective study design and the utilization of samples acquired at other centers would certainly contribute to further improving generalizability of results. However, low variability of results across different validation sets suggests sufficient robustness for assessing general feasibility and limitations of machine learning-based detection of early ischemic changes. Furthermore, the conducted permuted cross-validation allows valid performance assessments for the underlying dataset and its scanner-, protocol-, and hospital-specific image characteristics without overfitting effects. Statistically significant results with narrow confidence intervals hence indicate that the proposed algorithm could achieve similar metrics on NCCT images acquired within the same setup. In practice, hospital-specific customization and performance optimization of algorithms must be weighed against generalizability of classification results across different protocols, scanners, hospitals, or even modalities. In line with that, many of today’s FDA-certified artificial intelligence-based algorithms require a training period at the site to learn and integrate the respective site-specific characteristics. Second, the algorithm was trained to detect early signs of ischemia based on the presence of final infarction in 24–48 h FU NCCT images. Respective ground truth definition might suffer from limited sensitivity for small lesions and bias due to potential new hypoxic areas that form after admission imaging. However, assessments of infarction in 24–48 h follow-up NCCT imaging have been used as established imaging endpoints in numerous large clinical trials and hence are generally accepted as valid ground truth definition.

Third, the algorithm was trained to detect early ischemic changes in NCCT images at admission that offer limited sensitivity for detecting ischemia compared to DWI or PWI imaging. However, both scores—conventional ASPECTS and pc-ASPECTS—are originally based on evaluations of acute NCCT scans. NCCT scans at admission are fast and the technique is available in most hospitals. Furthermore, NCCT imaging is a fundamental part of most standard-of-care stroke protocols. Hence, a more sensitive machine learning-based detection of ischemic lesions in NCCT scans could allow for improved decision-making and prioritization concerning additional imaging diagnostics (e.g. MRI DWI/ADC) or therapeutic interventions. Fourth, the study cohort did not include any patients with old ischemic lesions in the posterior circulation. This could result in misclassifications of old lesions. However, if also trained on patients with old lesions, we would assume adequate class differentiation as early ischemia and old lesions express visually distinguishable imaging patterns. Fifth, the acquisition resolution of NCCT scans was limited to < 5 mm in slice thickness. The utilization of higher resolution images could improve classification performance. Sixth, only patients that underwent successful recanalization were included in the analysis. This might reduce generalizability of results as specific therapy effects modulate the development of infarct growth and imaging characteristics between admission and FU imaging. Seventh, the study cohort comprises patients with posterior circulation ischemia due to basilar artery occlusion only. This might cause potential model bias due to basilar occlusion-specific imaging characteristics within the analyzed pc-ASPECTS regions. However, pc-ASPECTS is not tied to any specific etiology; the score defines ROIs whose imaging-based assessment of early signs of ischemia has been shown to have high predictive power in forecasting patient outcome. The proposed algorithm evaluates quantitative image features of these regions without any utilization of etiology-related information. Hence, it can be assumed that the proposed algorithm may achieve similar results in patients with posterior circulation ischemia due to other etiologies. Eighth, the manual definition of pc-ASPECTS areas still implies a certain degree of observer dependence within the machine learning process. To minimize its influence, we derived standard maps from delineations obtained from 63 healthy subjects. Further, it was shown that radiomic features are comparably stable with regards to variations in segmentations [16, 27]. Ninth, only NCCT images were used for infarct prediction. Although computed tomographic perfusion imaging (CTP) may improve detection rates of posterior circulation ischemia [23], CTP images are difficult to interpret and can lead to false positive results [25]. Furthermore, the limitation to NCCT scans improves general applicability of the algorithm in clinical routine and retrospective studies, as not all hospitals perform CTP imaging in respective acute situations.

The proposed artificial intelligence-based algorithm confirms feasibility of automated, reproducible, and reader-independent detection of ischemic lesions in posterior circulation stroke patients. The predictive performance was superior or at least equal to visual assessments of two neuroradiologists. The proposed approach augments conventional readings by (a) integrating texture- and filter-based image features not assessable by human eyes and (b) employing artificial intelligence algorithms for automated and standardized data interpretation. The system may thereby facilitate reproducible analysis in future research and present an assisting tool for clinical decision-making, therapy planning and outcome prediction.

## Electronic supplementary material

Below is the link to the electronic supplementary material.

Supplementary file1 (XLSX 9624 kb)

## Data Availability

The datasets generated during and/or analysed during the current study are available from the corresponding author on reasonable request.
